# Integrating AMR surveillance into wastewater monitoring systems in 2025: a position on the implementation of Article 17 of the Urban Wastewater Treatment Directive (UWWTD)

**DOI:** 10.2807/1560-7917.ES.2026.31.3.2500289

**Published:** 2026-01-22

**Authors:** Louise Hock, Roosmarijn Luiken, Elisabete Valério, Marta Vargha, Julia Vierheilig, Stefan Börjesson, Tarja Pitkänen, Heike Schmitt

**Affiliations:** 1 Luxembourg Institute of Science and Technology (LIST), Environmental and Industrial Biotechnologies Unit, Belvaux, Luxembourg; 2 National Institute for Public Health and the Environment (RIVM), Bilthoven, The Netherlands; 3 Department of Environmental Health, National Institute of Health Doutor Ricardo Jorge, Lisboa, Portugal; 4 Department of Public Health Laboratories and Methodology, National Center for Public Health and Pharmacy, Budapest, Hungary; 5 TU Wien, Institute of Water Quality and Resource Management E226/1, Vienna, Austria; 6 Interuniversity Cooperation Centre Water & Health, Vienna, Austria; 7 Norwegian Veterinary Institute, Department of Animal Health, Welfare and Food Safety, Ås, Norway; 8 Finnish Institute for Health and Welfare, Department of Public Health, Kuopio, Finland; 9 University of Helsinki, Faculty of Veterinary Medicine, Helsinki, Finland; 10 Delft University of Technology, Delft, Netherlands; * These authors contributed equally to this work and share first/last authorship.

**Keywords:** Antimicrobial resistance (AMR), wastewater, wastewater surveillance, wastewater-based epidemiology, One Health

## Abstract

The recast Urban Wastewater Treatment Directive (UWWTD) calls for monitoring antimicrobial resistance (AMR) in wastewater of large European agglomerations (≥ 100,000 person equivalents). Guidance on scope and methods is currently in development. Two European Joint Actions share a goal to harmonise procedures and indicators: the European Union (EU)-Wastewater Integrated Surveillance for Public Health (EU-WISH), aiming to strengthen wastewater-based surveillance (WBS) for public health and the EU-Joint Action Antimicrobial Resistance and Healthcare Associated Infections (EU-JAMRAI) 2, providing among others, approaches for environmental surveillance of AMR. An EU-WISH survey in 2024, mapping WBS AMR-related activities across Europe, revealed that of 27 countries surveyed, 11 had an operative AMR WBS system and mainly employed WBS to determine AMR trends, primarily through culture-based analyses, in-depth characterisation of specific bacteria, and quantitative PCR for specific resistance genes. Occasionally metagenomics was used. We argue that prioritising AMR WBS targets should consider the intended objectives of surveillance, which could include uncovering AMR trends and emerging AMR determinants in humans, the assessment of antimicrobial/AMR environmental release, and wastewater treatment efficiency. Targets should be assessed for their public health relevance and the usefulness of complementary information they provide, while integrating measurability, resource efficiency, and expertise from different One Health domains.

## Background

The widespread use and misuse of antimicrobial agents have driven the emergence and spread of antimicrobial resistance (AMR), posing a challenge to modern societies. With at least 4.95 million deaths worldwide associated with drug-resistant bacterial infections in 2019 [1], AMR is considered an emerging pandemic and increasingly a global health crisis. The environment remains an under-addressed setting of AMR within the One Health continuum. However, it plays critical roles in AMR: as a reservoir hosting resistant bacteria and resistance genes from human and animal waste, as a source of resistance mechanisms, and as a medium for resistance transmission back to humans and animals [2]. Efforts of environmental surveillance often target wastewater treatment plants (WWTPs) as important entry points of AMR into the environment.

Additionally, wastewater-based surveillance (WBS) has recently gained prominence as a complementary approach to clinical surveillance of human infections, primarily for tracking circulation of viruses (e.g. severe acute respiratory syndrome coronavirus 2 (SARS-Cov-2)), but also for monitoring AMR. This approach uses wastewater analysis to track pathogens and resistance patterns at population level. Unlike clinical surveillance that primarily captures symptomatic cases, WBS non-invasively monitors entire communities including asymptomatic carriers, while also contributing to track environmental contamination by AMR-carrying organisms, antimicrobial resistance genes (ARGs) and antimicrobials.

In the recast of the European urban wastewater treatment directive (UWWTD) [3], the recent inclusion of AMR monitoring within urban wastewater surveillance (Article 17) requires harmonisation of WBS approaches within Europe. This aligns with calls for harmonised environmental surveillance issued by inter alia*,* the European Union (EU) [4] and the United Nations General Assembly [5]. In this paper, we present findings from the EU-Wastewater Integrated Surveillance for Public Health (EU-WISH) Joint Action (https://www.eu-wish.eu/about-us/mission−/−vision) survey on existing AMR WBS activities across European countries and propose a structured framework for establishing effective AMR WBS systems in the future.

## AMR surveillance harmonisation activities for UWWTD Article 17 implementations

Under the recast UWWTD [3], EU countries will be required to monitor AMR in urban WWTPs of agglomerations with ≥ 100,000 person equivalents (Article 17). The scope of this obligation and means to comply to it are currently being defined and developed, and guidance for AMR WBS is required for standardisation, quality control, interpretation, ethical considerations, resource allocation, and integration with other surveillance systems [6]. Capacity building is also needed to avoid inconsistency, unreliable data, or failure to translate findings into meaningful public health actions.

So far, AMR WBS has mainly been addressed in scientific research projects [7-11] and pilot actions preparing for the forthcoming mandatory monitoring of AMR in wastewater [12]. In addition, several collaborative initiatives have recently started developing guidance for environmental surveillance of AMR including WBS. The World Health Organization (WHO) Tricycle protocol proposes integrated surveillance protocols for a single indicator, extended spectrum β-lactamase (ESBL) producing *Escherichia coli*, i.e. in wastewater and surface water [13]. Under the umbrella of the European Environmental Agency within the European Environment Information and Observation Network (EIONET) [14], a recent international collaboration established and piloted a methodology for monitoring antibiotic resistance in aquatic environments [14].

In terms of other future and ongoing initiatives, an Ad hoc Group ‘Antimicrobial resistance’ (AHG AMR) will be established by the subcommittee SC4 under the Technical Committee 147 of the International Organization for Standardization (ISO) to prepare a new proposal to develop an ISO method for determining AMR in water. In addition, two EU Joint Actions, which are initiatives co-funded by the European Commission and EU countries to deal with health-related priorities requiring action at the European level, can contribute to guidance for obligatory AMR WBS. The first is the Joint Action Antimicrobial Resistance and Healthcare-Associated Infections (EU-JAMRAI 2), which also works towards defining objectives, sampling schemes and indicators for surveillance of AMR in the wider environment including wastewater (https://eu-jamrai.eu/). The second is EU-WISH, which addresses implementation options of multiple pathogen targets for WBS, among them AMR.

## Current state of AMR wastewater-based surveillance in Europe

One of the first actions of the EU-WISH consortium was conducting a survey to map existing WBS activities in 2024, also regarding AMR, across Europe. Twenty-six European partner countries (https://www.eu-wish.eu/about-us/partners) and one invited European country were asked to gather the national information required to conduct the survey.

**Figure 1:**
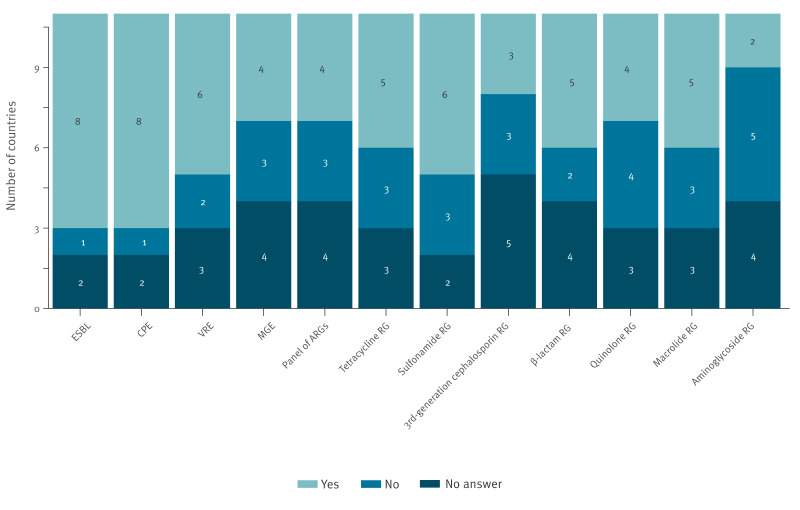
Targets of AMR wastewater-based surveillance in Europe according to the EU-WISH survey, 2024 (n = 11 participating countries)

AMR: antimicrobial resistance; ARGs: antimicrobial resistance genes; CPE: carbapenemase-producing Enterobacterales; ESBL: extended spectrum β-lactamase producing *Escherichia coli*; EU-WISH: European Union Wastewater Integrated Surveillance for Public Health; MGE: mobile genetic element; RG: resistance gene; VRE: vancomycin-resistant enterococci; WBS: wastewater-based surveillance.

Of the 27 countries surveyed, 11 reported having an operative AMR WBS system (defined as programmes implemented either as research or national public health initiatives, whether of limited duration or institutionalised, regardless of data being collected in an exploratory phase or being reported to key decisionmakers). These 11 countries spanned various regions of Europe, including Northern, Southern, and Central Europe. The reported objectives of AMR WBS were to identify trends in AMR (8/11), early detection of emerging AMR (3/11) and genotyping of antimicrobial-resistant strains (3/11). The main targets, in terms of bacteria, were ESBL-producing Enterobacterales and carbapenemase-producing Enterobacterales (CPE), followed by vancomycin-resistant enterococci (VRE) and, in terms of ARGs, sulfonamide-resistance genes (Figure 1).

Data, collected through the EU-WISH Joint Action survey, originated from 11 countries among the 27 countries, which were invited to participate in the survey. These 11 countries had answered ‘yes’ to the question: ‘Do you currently perform of did you perform in previous years WBS for AMR?’.

**Figure 2:**
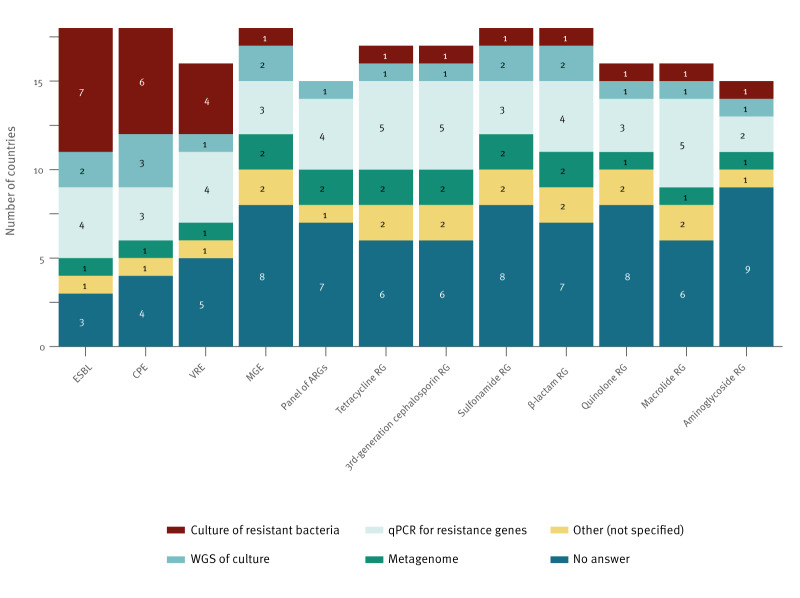
AMR WBS detection techniques per target according to the EU-WISH survey, 2024 (n = 11 participating countries)

AMR: antimicrobial resistance; ARGs: antimicrobial resistance genes; CPE: carbapenemase-producing Enterobacterales; ESBL: extended spectrum β-lactamase producing *Escherichia coli*; EU-WISH: European Union Wastewater Integrated Surveillance for Public Health; MGE: mobile genetic element; RG: resistance gene; VRE: vancomycin-resistant enterococci; WBS: wastewater-based surveillance; WGS: whole-genome sequencing; qPCR: quantitative PCR.

To detect resistant bacteria, culture-based techniques were most frequently employed, while quantitative (q)PCR techniques were the main method to detect ARGs (Figure 2).

Data, collected via the EU-WISH Joint Action survey, originated from 11 countries among the 27 countries, which were invited to participate in the survey. These 11 countries had answered ‘yes’ to the questions: ‘Do you currently perform of did you perform in previous years WBS for AMR? What techniques are used?’. Multiple answers were possible.

## Process towards harmonised surveillance of AMR in wastewater

**Figure 3:**
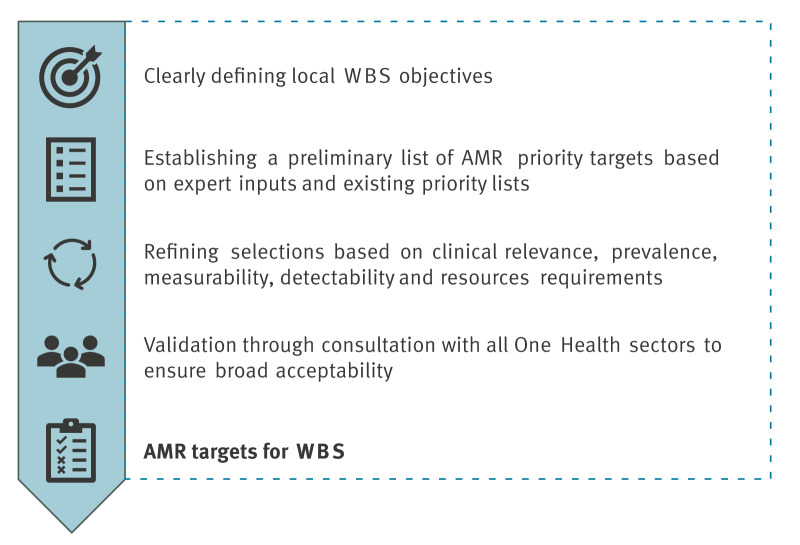
Key steps for establishing a list of priority AMR targets for WBS, 2024

AMR: antimicrobial resistance; WBS: wastewater-based surveillance.

Currently, there is no consensus list of priority AMR targets for WBS or harmonised procedure for AMR target prioritisation. A transparent prioritisation framework would enable the creation of a priority AMR target list that can be adapted across time and locations. The Joint Actions EU-WISH and EU-JAMRAI 2 are developing such a framework based on the WHO pathogen priority list [15], WHO guidance for WBS pathogen prioritisation, and other previously used prioritisation protocols such as those of the Registration, Evaluation, Authorisation and Restriction of Chemicals (REACH) for chemical substances. Though still under development, the authors recommend incorporating the following key aspects (Figure 3): (i) clearly defining local WBS objectives; (ii) establishing a preliminary list of AMR priority targets based on expert inputs and existing priority lists (WHO bacterial pathogens list, national surveillance); (iii) refining selections based on clinical relevance, prevalence, measurability, detectability and resources requirements; (iv) validation through consultation with all One Health sectors (e.g. public health, animal health, health of the environment) to ensure broad acceptability.

This process will lead to a ranking of AMR targets for WBS that can be updated with new evidence or emerging AMR threats. Harmonisation must also address sampling frequency and population coverage to facilitate interpretation and integration with other surveillance systems.

## Objectives of AMR wastewater-based surveillance

Wastewater analysis serves dual purposes: it provides insights into AMR patterns within the population while also measuring AMR discharge into aquatic ecosystems, enabling multiple objectives for AMR WBS. Specific objectives of AMR WBS, conducted through monitoring untreated wastewater (influent) of WWTPs consist of (i) establishing temporal trends in the occurrence of specific genetic determinants of resistance and (multidrug-)resistant pathogens, their variations, as well as temporal trends of antimicrobial residues in or from the targeted community, and (ii) alerting to new forms of AMR emerging in the community (early detection). These objectives are highly similar to those of human clinical surveillance, and to the aims of WBS applied to pathogens that are monitored for other purposes than AMR. If WBS is conducted at smaller scale (i.e. healthcare institutions), it might also assist in outbreak detection.

Monitoring treated wastewater (effluent) of WWTPs can contribute to evaluating the role of the environment as AMR transmission route. Data generated from effluent monitoring (iii) help to conduct risk or exposure assessments of humans and animals, e.g. via emissions from WWTPs to surface water, reused wastewater, and the related risks for recreational and agricultural exposure, and (iv) helps in assessing hazardous environmental impacts of antimicrobials and other trace pollutants, particularly their role in resistance selection and driving AMR evolution.

Last, by monitoring both influent and effluent, (v) the efficiency and impact of WWTP treatment for the reduction of AMR (and antimicrobials) can be assessed, including the impact of investments in advanced WWTP techniques.

## Fulfilling wastewater-based objectives through purpose-driven AMR target selection

The different objectives for WBS require a strategic choice of targets (Table), and, unfortunately, a single indicator cannot serve all objectives. If the objective of WBS consists of a complementary approach to human clinical (or veterinary) epidemiology and surveillance (objectives i and ii), the harmonisation of targets with those under human and animal surveillance is important for integrated surveillance. Implementation of culture-based methods enable harmonisation with current ongoing EU surveillance such as that conducted by the European Antimicrobial Resistance Surveillance Network (EARS-Net; https://www.ecdc.europa.eu/en/about-us/networks/disease-networks-and-laboratory-networks/ears-net-data), the European Food Safety Authority (EFSA) and the European Antimicrobial Resistance Surveillance Network in Veterinary Medicine (EARS-Vet; https://eu-jamrai.eu/surveillance/ears-vet/). One of the main advantages of WBS is its sensitivity towards detecting low prevalence antibiotic resistant bacteria carried by asymptomatic individuals [11,16]. With the objective to follow trends in population carriage, WBS would offer added value for surveillance of resistant microorganisms of high public health relevance (e.g. WHO priority list and/or national surveillance) and with low community carriage (e.g. highlighted in national surveillance and population studies). This surveillance objective is exemplified by CPE, which is a key target reported in the EU-WISH survey.

**Table:** General overview of ideal characteristics of targets and methods to fulfil specific objectives of AMR surveillance at community wastewater treatment plants, 2024

Target and methods characteristics		Objectives of monitoring				
		WWTP influent		WWTP effluent		WWTP efficiency
		(i) AMR trends in community	(ii) Early detection of emerging AMR	(iii) AMR emissions^a^	(iv) Antibiotic residue emissions^a^	(v) AMR reduction efficiency
Targets	Human public health relevant	**Relevant**	**Relevant**	**Relevant**	**Relevant**	LR
	Animal health relevant	**Relevant**	**Relevant**	**Relevant**	**Relevant**	LR
	Environmental health relevant	LR	LR	**Relevant**	**Relevant**	LR
	Regularly detectable	LR	LR	**Relevant**	**Relevant**	**Relevant**
Methods	Culture-based	**Relevant**	**Relevant**	**Relevant**	LR	**Relevant**
	PCR-based	**Relevant**	LR	**Relevant**	LR	**Relevant**
	Metagenomic sequencing	**Relevant**	**Relevant**	**Relevant**	LR	LR
	LC-MS	LR	LR	LR	**Relevant**	LR

AMR: antimicrobial resistance; LC-MS: liquid chromatography mass spectrometry; LR: less relevant; WWTP: wastewater treatment plant.

^a^ Emissions via WWTP effluent to surface water.

Relevant targets are in bold font.

With respect to culture-based targets, whole genome sequencing (WGS) identifies genetic variants, while parallel monitoring of a denominator such as *E. coli*, establishes trends in relative AMR abundance. Amplification-based molecular methods, such as qPCR or digital PCR (dPCR), could also be used to reach objectives, i.e. by allowing a targeted quantification of specific genetic determinants of resistance. However, they are limited to previously characterised target genes and do not provide information on the host species or location on mobile genetic elements [17,18], making the interpretation of data for public health more difficult. Metagenomic sequencing provides a non-targeted approach for WBS, theoretically capturing all genetic information to provide a comprehensive profile of the resistome and discover potential new ARGs [19] (objective ii). Data can be reanalysed when novel resistance mechanisms emerge, and analysis can extend beyond AMR to other bacterial and viral pathogens. However, short-read sequencing offers limited information into the association between resistance genes, mobile genetic elements, and hosts [20]. This might partially be overcome by long-read sequencing, but the sequencing depth may affect the detection of rare ARGs and variants. The EU-WISH survey results indicate that metagenomic application in AMR WBS has begun, with sufficient research expertise available to support wider implementation.

If the objective is to evaluate WWTP effluent emissions of AMR to surface water (objective iii), targets should be of public or animal health relevance, but also sufficiently common to be regularly detected, again possibly in combination with general faecal indicators like *E. coli* or with human-associated microbial source tracking (MST) markers. However, for a more complete evaluation of AMR emissions from WWTPs, also utilisation of wastewater sludge should be considered. Objective iv calls for monitoring residues of antimicrobials and possibly other chemicals (e.g. antifungals or heavy metals). This aligns with the European Water Framework Directive, which aims to maintain good surface water quality and includes monitoring of various antibiotics residues in surface water [21] to protect environmental health and safeguard ecosystems from anthropogenic adverse impacts. In contrast to the potential impact of such residues on environmental health, the primary concern regarding AMR is its impact on humans and animals undergoing antibiotic treatment.

To evaluate the efficiency of wastewater treatment (objective v), a wide choice of targets that offer quantitative data are possible, ranging from specific resistant or non-resistant bacteria to quantitative measurements of resistance genes through qPCR/digital (d)PCR. However, these molecular methods can also detect desoxyribonucleic acid (DNA) from non-viable organisms, extracellular DNA and intrinsic resistance genes, which are all less relevant to public health but should not be neglected [22,23]. For discrimination between viable and non-viable organisms, viability PCR methods could be applied [24].

Target selection for AMR surveillance requires interdisciplinary collaboration between environmental microbiologists, experts in surveillance and control of human and animal AMR, and other stakeholders. The targets may vary by country based on local AMR prevalence patterns. Effective AMR WBS requires standardised protocols, like ISO standards, across all stages – from sampling through (bioinformatic) analysis – to ensure results are robust, reproducible, and comparable. Quality assurance/control systems must be implemented, and establishing dedicated reference laboratories at national and European levels should be considered to support these efforts. The advantages and limitations of each method have already been discussed in previous studies [25,26].

## Conclusions

European experience demonstrates that AMR WBS is both feasible and increasingly implemented (data not shown), making it valuable for the UWWTD. AMR WBS can serve multiple purposes: tracking AMR circulation in populations, identifying emerging variants (with new antimicrobial resistance determinants), and measuring AMR emissions from WWTP effluents into the environment. As comprehensive monitoring is economically impractical, AMR targets should be strategically selected based on specific objectives. Both EU-WISH and EU-JAMRAI 2 are committed to harmonising AMR WBS methodologies across Europe, facilitating the efficient implementation and integration of this surveillance system into a cohesive European One Health monitoring framework.

## Data Availability

The data used in this study are available as Supplementary Table S1.

## Supplementary Data

72000 Supplementary Table S1

## References

[1] Murray CJ Ikuta KS Sharara F Swetschinski L Robles Aguilar G Gray A Antimicrobial Resistance Collaborators. Global burden of bacterial antimicrobial resistance in 2019: a systematic analysis. Lancet. 2022;399(10325):629-55. 10.1016/S0140-6736(21)02724-0 35065702 PMC8841637

[2] Larsson DGJ Flach CF. Antibiotic resistance in the environment. Nat Rev Microbiol. 2022;20(5):257-69. 10.1038/s41579-021-00649-x 34737424 PMC8567979

[3] The European Parliament and the council of the European Union. Directive (EU) 2024/3019 of the European parliament and of the council of 27 November 2024 concerning urban wastewater treatment (recast). Official Journal of the European Union. 12.12.2024.L 2024/3019. Available from: https://eur-lex.europa.eu/legal-content/EN/TXT/?uri=CELEX%3A32024L3019&qid=1762354065172

[4] European Commission. A European One Health Action Plan against AMR. Brussels: European Commission; 2017. Available from: https://health.ec.europa.eu/system/files/2020-01/amr_2017_action-plan_0.pdf

[5] United Nations General Assembly. Political Declaration of the High-level Meeting on Antimicrobial Resistance. 2024.

[6] Conforti S Pruden A Acosta N Anderson C Buergmann H Calabria De Araujo J Strengthening policy relevance of wastewater-based surveillance for antimicrobial resistance. Environ Sci Technol. 2025;59(5):2339-43. 10.1021/acs.est.4c09663 39874274 PMC11823445

[7] Tiwari A Lehto KM Paspaliari DK Al-Mustapha AI Sarekoski A Hokajärvi AM WastPan Study Group. Developing wastewater-based surveillance schemes for multiple pathogens: The WastPan project in Finland. Sci Total Environ. 2024;926:171401. 10.1016/j.scitotenv.2024.171401 38467259

[8] Tiwari A Krolicka A Tran TT Räisänen K Ásmundsdóttir ÁM Wikmark OG Antibiotic resistance monitoring in wastewater in the Nordic countries: A systematic review. Environ Res. 2024;246:118052. 10.1016/j.envres.2023.118052 38163547

[9] Hendriksen RS Munk P Njage P van Bunnik B McNally L Lukjancenko O Global Sewage Surveillance project consortium. Global monitoring of antimicrobial resistance based on metagenomics analyses of urban sewage. Nat Commun. 2019;10(1):1124. 10.1038/s41467-019-08853-3 30850636 PMC6408512

[10] Hultman J Tamminen M Pärnänen K Cairns J Karkman A Virta M. Host range of antibiotic resistance genes in wastewater treatment plant influent and effluent. FEMS Microbiol Ecol. 2018;94(4):fiy038. 10.1093/femsec/fiy038 29514229 PMC5939699

[11] Blaak H Kemper MA de Man H van Leuken JPG Schijven JF van Passel MWJ Nationwide surveillance reveals frequent detection of carbapenemase-producing Enterobacterales in Dutch municipal wastewater. Sci Total Environ. 2021;776:145925. 10.1016/j.scitotenv.2021.145925

[12] Benedetti G Wulff Krogsgaard L Maritschnik S Stüger HP Hutse V Janssens R A survey of the representativeness and usefulness of wastewater-based surveillance systems in 10 countries across Europe in 2023. Euro Surveill. 2024;29(33):1-11. 10.2807/1560-7917.ES.2024.29.33.2400096 39149824 PMC11328501

[13] World Health Organization (WHO). WHO integrated global surveillance on ESBL-producing E. coli using a ‘One Health’ approach: implementation and opportunities. Geneva: WHO; 2021. Available from: https://www.who.int/publications/i/item/9789240021402

[14] Schwermer CU Krzeminski P Anglès d’Auriac M Gjeitnes M Moe J Bellanger X Pilot study on antimicrobial resistance monitoring in European surface waters - Final report of the Eionet Working Group. Zenodo. 2025.

[15] World Health Organization (WHO). Bacterial Priority Pathogens List, 2024: bacterial pathogens of public health importance to guide research, development and strategies to prevent and control antimicrobial resistance. Geneva: WHO; 2024. Available from: https://www.who.int/publications/i/item/9789240093461

[16] Böttcher S Kreibich J Wilton T Saliba V Blomqvist S Al-Hello H Detection of circulating vaccine-derived poliovirus type 2 (cVDPV2) in wastewater samples: a wake-up call, Finland, Germany, Poland, Spain, the United Kingdom, 2024. Euro Surveill. 2025;30(3):2-9. 10.2807/1560-7917.ES.2025.30.3.2500037 39850005 PMC11914958

[17] Huijbers PMC Bobis Camacho J Hutinel M Larsson DGJ Flach CF. Sampling Considerations for Wastewater Surveillance of Antibiotic Resistance in Fecal Bacteria. Int J Environ Res Public Health. 2023;20(5):4555. 10.3390/ijerph20054555 36901565 PMC10002399

[18] Zhou S Lou EG Schedler J Ensor KB Hopkins L Stadler LB. Comparative analysis of culture- and ddPCR-based wastewater surveillance for carbapenem-resistant bacteria. Environ Sci Water Res Technol. 2024;11(1):51-63. 10.1039/D4EW00525B

[19] Berglund F Österlund T Boulund F Marathe NP Larsson DGJ Kristiansson E. Identification and reconstruction of novel antibiotic resistance genes from metagenomes. Microbiome. 2019;7(1):52. 10.1186/s40168-019-0670-1 30935407 PMC6444489

[20] Lou EG Fu Y Wang Q Treangen TJ Stadler LB. Sensitivity and consistency of long- and short-read metagenomics and epicPCR for the detection of antibiotic resistance genes and their bacterial hosts in wastewater. J Hazard Mater. 2024;469:133939. 10.1016/j.jhazmat.2024.133939 38490149

[21] Proposal for a Directive of the European Parliament and of the Council amending Directive 2000/60/EC establishing a framework for Community action in the field of water policy, Directive 2006/118/EC on the protection of groundwater against pollution and deterioration and Directive 2008/105/EC on environmental quality standards in the field of water policy - Revised Presidency compromise text. Brussels: European Commission; 2022. Available from: https://eur-lex.europa.eu/legal-content/EN/TXT/?uri=CELEX:52022PC0540

[22] Woegerbauer M Bellanger X Merlin C. Cell-Free DNA: An Underestimated Source of Antibiotic Resistance Gene Dissemination at the Interface Between Human Activities and Downstream Environments in the Context of Wastewater Reuse. Front Microbiol. 2020;11:671. 10.3389/fmicb.2020.00671 32390973 PMC7192050

[23] Oliveira M Nunes M Barreto Crespo MT Silva AF. The environmental contribution to the dissemination of carbapenem and (fluoro)quinolone resistance genes by discharged and reused wastewater effluents: The role of cellular and extracellular DNA. Water Res. 2020;182:116011. 10.1016/j.watres.2020.116011 32623198

[24] Eramo A Morales Medina WR Fahrenfeld NL. Viability-based quantification of antibiotic resistance genes and human fecal markers in wastewater effluent and receiving waters. Sci Total Environ. 2019;656:495-502. 10.1016/j.scitotenv.2018.11.325 30522032 PMC6526933

[25] Clarke LM O’Brien JW Murray AK Gaze WH Thomas KV. A review of wastewater-based epidemiology for antimicrobial resistance surveillance. J Environ Expo Assess. 2024;3(1). 10.20517/jeea.2023.29

[26] Klümper U, Leonard AFC, Stanton IC, Anjum MF, Antonio M, Balkhy H, et al. Towards developing an international AMR surveillance strategy. Joint Programming Initiative on Antimicrobial Resistance (JPIAMR); 2022. Available from: https://jpiamr.eu/app/uploads/2022/08/Towards-developing-an-international-environmental-AMR-surveillance-strategy_report-2022-08-04.pdf

