# Supplementary material for: Integrating AMR surveillance into wastewater monitoring systems in 2025: a position on the implementation of Article 17 of the Urban Wastewater Treatment Directive (UWWTD)
Source: Euro Surveill. 2026 Jan 22;31(3):2500289. doi: 10.2807/1560-7917.ES.2026.31.3.2500289 (PMC12848984; doi:10.2807/1560-7917.ES.2026.31.3.2500289)
Supplement: Supplementary Table S1 [file 2500289_SupplementaryTableS1.pdf]

Supplementary Table S1: Data collected from the EU-WISH survey mapping existing WBS activities across Europe

This supplementary material is hosted by *Eurosurveillance* as supporting information alongside the article Integrating AMR surveillance into wastewater monitoring systems in 2025: a position on the implementation of Article 17 of the Urban Wastewater Treatment Directive (UWWTD), on behalf of the authors, who remain responsible for the accuracy and appropriateness of the content. The same standards for ethics, copyright, attributions and permissions as for the article apply. Supplements are not edited by *Eurosurveillance* and the journal is not responsible for the maintenance of any links or email addresses provided therein.

**Country**	**Do you have an operative WES system for AMR in your country in 2024?**	**Do you currently perform or did you perform in previous years WES for AMR? : ESBL (beta-lactamase E. coli) : Detection techniques?**	**Do you currently perform or did you perform in previous years WES for AMR? : CPE (carbapenemase enterobacter) : Detection techniques?**	**Do you currently perform or did you perform in previous years WES for AMR? : VRE (vancomycin resistant Enterococcus) : Detection techniques?**	**Do you currently perform or did you perform in previous years WES for AMR? : Plasmids or mobile genetic elements : Detection techniques?**	**Do you currently perform or did you perform in previous years WES for AMR? : Panel of Resistance genes : Detection techniques?**	**Do you currently perform or did you perform in previous years WES for AMR? : tetracycline resistant genes : Detection techniques?**	**Do you currently perform or did you perform in previous years WES for AMR? : sulfonamide resistant genes : Detection techniques?**	**Do you currently perform or did you perform in previous years WES for AMR? : 3rd-generation cephalosporin resistant genes : Detection techniques?**	**Do you currently perform or did you perform in previous years WES for AMR? : Beta-lactam resistance genes : Detection techniques?**	**Do you currently perform or did you perform in previous years WES for AMR? : Quinolone resistance genes : Detection techniques?**	**Do you currently perform or did you perform in previous years WES for AMR? : Macrolide resistance genes : Detection techniques?**	**Do you currently perform or did you perform in previous years WES for AMR? : Aminoglycoside resistance genes : Detection techniques?**
Country1	No	No answer	No answer	No answer	No answer	No answer	No answer	No answer	No answer	No answer	No answer	No answer	No answer
Country2	No	No answer	No answer	No answer	No answer	No answer	No answer	No answer	No answer	No answer	No answer	No answer	No answer
Country3	No	No answer	No answer	No answer	No answer	No answer	No answer	No answer	No answer	No answer	No answer	No answer	No answer
Country4	Yes	Culture of resistant bacteria	Culture of resistant bacteria; WGS of culture	Culture of resistant bacteria	No answer	No answer	qPCR for resistance genes	qPCR for resistance genes	No answer	No answer	qPCR for resistance genes	qPCR for resistance genes	No answer
Country5	No	No answer	No answer	No answer	No answer	No answer	No answer	No answer	No answer	No answer	No answer	No answer	No answer
Country6	Yes	No answer	No answer	No answer	No answer	No answer	No answer	No answer	No answer	No answer	No answer	No answer	No answer
Country7	No	No answer	No answer	No answer	No answer	No answer	No answer	No answer	No answer	No answer	No answer	No answer	No answer
Country8	No	No answer	No answer	No answer	No answer	No answer	No answer	No answer	No answer	No answer	No answer	No answer	No answer
Country9	Yes	Culture of resistant bacteria; WGS of culture	Culture of resistant bacteria; WGS of culture	No answer	WGS of culture; qPCR for resistance genes; Metagenome; Other	WGS of culture; qPCR for resistance genes; Metagenome; Other	qPCR for resistance genes; Metagenome; Other	qPCR for resistance genes; Metagenome; Other	WGS of culture; qPCR for resistance genes; Metagenome; Other	WGS of culture; qPCR for resistance genes; Metagenome; Other	qPCR for resistance genes; Other	qPCR for resistance genes; Other	No answer
Country10	Yes	Culture of resistant bacteria; qPCR for resistance genes	Culture of resistant bacteria; qPCR for resistance genes	Culture of resistant bacteria; qPCR for resistance genes	qPCR for resistance genes	qPCR for resistance genes; Metagenome	qPCR for resistance genes	qPCR for resistance genes	qPCR for resistance genes	qPCR for resistance genes	No answer	qPCR for resistance genes	No answer
Country11	No	No answer	No answer	No answer	No answer	No answer	No answer	No answer	No answer	No answer	No answer	No answer	No answer
Country12	No	No answer	No answer	No answer	No answer	No answer	No answer	No answer	No answer	No answer	No answer	No answer	No answer
Country13	Yes	Culture of resistant bacteria; WGS of culture; qPCR for resistance genes; Metagenome; Other	Culture of resistant bacteria; WGS of culture; qPCR for resistance genes; Metagenome; Other	Culture of resistant bacteria; WGS of culture; qPCR for resistance genes; Metagenome; Other	Culture of resistant bacteria; WGS of culture; qPCR for resistance genes; Metagenome; Other	No answer	Culture of resistant bacteria; WGS of culture; qPCR for resistance genes; Metagenome; Other	Culture of resistant bacteria; WGS of culture; qPCR for resistance genes; Metagenome; Other	Culture of resistant bacteria; WGS of culture; qPCR for resistance genes; Metagenome; Other	Culture of resistant bacteria; WGS of culture; qPCR for resistance genes; Metagenome; Other	Culture of resistant bacteria; WGS of culture; qPCR for resistance genes; Metagenome; Other	Culture of resistant bacteria; WGS of culture; qPCR for resistance genes; Metagenome; Other	Culture of resistant bacteria; WGS of culture; qPCR for resistance genes; Metagenome; Other
Country14	No	No answer	No answer	No answer	No answer	No answer	No answer	No answer	No answer	No answer	No answer	No answer	No answer
Country15	Yes	No answer	No answer	No answer	No answer	No answer	No answer	No answer	No answer	No answer	No answer	No answer	No answer
Country16	No	No answer	No answer	No answer	No answer	No answer	No answer	No answer	No answer	No answer	No answer	No answer	No answer
Country17	No	No answer	No answer	No answer	No answer	No answer	No answer	No answer	No answer	No answer	No answer	No answer	No answer
Country18	Yes	Culture of resistant bacteria	Culture of resistant bacteria	No answer	No answer	qPCR for resistance genes	No answer	No answer	No answer	No answer	No answer	No answer	No answer
Country19	No	No answer	No answer	No answer	No answer	No answer	No answer	No answer	No answer	No answer	No answer	No answer	No answer
Country20	Yes	qPCR for resistance genes	qPCR for resistance genes	qPCR for resistance genes	No answer	qPCR for resistance genes	No answer	No answer	No answer	qPCR for resistance genes	No answer	No answer	No answer
Country21	No	No answer	No answer	No answer	No answer	No answer	No answer	No answer	No answer	No answer	No answer	No answer	No answer
Country22	Yes	No answer	No answer	No answer	No answer	No answer	No answer	No answer	No answer	No answer	No answer	No answer	No answer
Country23	No	No answer	No answer	No answer	No answer	No answer	No answer	No answer	No answer	No answer	No answer	No answer	No answer
Country24	Yes	Culture of resistant bacteria	Culture of resistant bacteria	Culture of resistant bacteria	No answer	No answer	No answer	No answer	No answer	No answer	No answer	No answer	No answer
Country25	Yes	Culture of resistant bacteria; qPCR for resistance genes	No answer	qPCR for resistance genes	No answer	No answer	qPCR for resistance genes	qPCR for resistance genes	No answer	No answer	No answer	qPCR for resistance genes	qPCR for resistance genes
Country26	No	No answer	No answer	No answer	No answer	No answer	No answer	No answer	No answer	No answer	No answer	No answer	No answer
Country27	No	No answer	No answer	No answer	No answer	No answer	No answer	No answer	No answer	No answer	No answer	No answer	No answer
